# Unmet Occupational Health Needs of Malawian Ex-Miners from the South African Gold Mines

**DOI:** 10.5334/aogh.4680

**Published:** 2025-06-05

**Authors:** Rinila Haridas, Rodney Ehrlich, Yotam Moyo, Annalee Yassi, Jerry Spiegel, Khumbo Kalua

**Affiliations:** 1 School of Population and Public Health, Faculty of Medicine, University of British Columbia, Vancouver, British Columbia, Canada; 2 Division of Occupational Medicine, School of Public Health, University of Cape Town, South Africa; 3 Department of Occupational and Health Services, Ministry of Health, Government of Malawi, Lilongwe, Malawi

**Keywords:** ex-gold miners, mining, social security, silicosis, tuberculosis, Malawi, South Africa

## Abstract

*Background:* From early in its history, gold mining in South Africa involved recruiting hundreds of thousands of workers from Malawi and other neighbouring countries to work in an environment conducive to high rates of tuberculosis and silicosis. Official recruitment from Malawi ended in the 1990s, depriving large numbers of these migrant miners of their livelihood, with limited or no access to employment-linked social benefits and services.

*Objectives:* To describe barriers faced by Malawian migrant ex-gold miners in accessing social benefits related to occupational lung disease and related health services and medical examinations, and to identify needed actions.

*Methods:* This study, conducted in the Blantyre region of Malawi, draws from field observations and interviews with 14 ex-gold miners who had worked on South African gold mines, supplemented by five key informant interviews. Data were analysed using a phenomenological and thematic analysis approach.

*Findings:* Ex-migrant miners described precarious livelihoods and difficulty accessing employment-linked benefit examinations and health services. They are largely uncertain about their entitlements related to their past work in South Africa and the means for pursuing such rights. The division of responsibility within South Africa and between the governments of South Africa and Malawi has resulted in confusion and misinformation. Within Malawi, scarcity of funding, administrative hurdles and limited occupational lung disease expertise are barriers to expanding current services for ex-migrant miners as well as ex-miners from Malawian mines.

*Conclusions:* A number of actions are needed: coordination between the Malawian government and South African agencies; integration of occupational health services, including for migrant ex-gold miners, into Malawi’s public health system; education of ex-gold miners and their dependents about their rights and related processes and the provision of relief aid through local and external support. Financial involvement of the South African mining industry that profited from the services of migrant miners is required to alleviate the burden on publicly funded health systems.

## Introduction

From the late 19th century, South Africa’s gold mining industry relied on a circular migrant labour system recruiting workers from rural communities in South Africa and neighbouring countries. By the 1960s and early 1970s, over half of the workers in the industry came from outside South Africa [1]. Peak employment was reached in the period 1983–1987, with 750,000 workers in the industry of whom a third were foreign [2]. While the countries of major recruitment were Mozambique and Lesotho, among these workers were migrants from Nyasaland (now Malawi), first recruited in 1903 [3].

Entrenched under colonial and apartheid regimes in Southern Africa, the migrant labour system was associated with low wages, crowded transport and hostel accommodation, exposure to sexually transmitted diseases and the hazardous environment of deep‑level mining. The last included severe safety risks: heat, humidity, noise and exposure to silica‑containing dust [4]. Throughout the 20th century, these conditions, in conjunction with the circular migration pattern of workers travelling between their home countries and South Africa, contributed to a public health crisis of occupational lung disease [5–7]. Mainly, this encompassed tuberculosis (TB) and silicosis, a fibrotic lung disease caused by silica dust inhalation that further increases the risk of TB [5–8]. TB in miners has a number of risk factors but is classified legally as an occupational disease in South Africa owing to the strong association of TB with silica exposure, with or without silicosis [8].

By the last quarter of the 20th century, a new labour recruitment regimen in the South African gold mines [9] and a simultaneous HIV epidemic had resulted in a triple epidemic of TB, silicosis and HIV affecting a number of migrant home regions, including South Africa, Lesotho, Mozambique, Eswatini and Botswana [5, 7, 10, 11]. While the prevalence of occupational lung disease in Malawian migrant ex‑miners is not known, they are likely to carry a risk of both silicosis and TB, including late occurring and recurrent TB linked to their years in the South African gold mines [7, 12]. However, a feature of the migrant labour system is that, upon returning home, surviving ex‑gold miners have had reduced access to benefits linked to their mining employment. While employment benefits encompass a number of potential entitlements, the focus of this study is on health services and compensation benefits for occupational lung disease.

A singular feature of the recruitment of Malawian migrant miners was that while most miners from the subcontinent were recruited via TEBA, the central recruiting agency of the South African mining industry, from 1967 onwards, the Malawi Government Department of Labour played a formal role in these processes in an arrangement with TEBA [13, 14]. By 1970, the peak year, 80,000 Malawian migrant miners were employed in South African mines [2].

This participation was tragically disrupted in 1974 when 74 Malawian migrant workers returning from South Africa died in a plane crash in Botswana, resulting in the then‑president of Malawi abruptly stopping the contract recruitment of migrant labour via TEBA. This action affected 129,000 Malawian migrant miners from northern and southern regions of Malawi [1, 15]. A lower rate of recruitment was subsequently reinstated until 1988, when the finding of HIV infection in screened Malawian recruits led to a South African government requirement for mandatory HIV testing prior to recruitment [15, 16]. Following refusal by the Malawian government, 13,000 workers were repatriated over the subsequent four years. TEBA recruitment operations in Malawi were closed in 1988 [14]. Although Malawians have continued to find employment (as ‘freelancers’) in the South African mining industry [14, 17], the events above profoundly affected the livelihoods of a large number of Malawians who had come to depend on migrant mining for their income. A 2020 estimate is that there were over 9,000 migrant ex‑miners in Malawi who had worked in South Africa until the early 1990s [14].

Despite recent initiatives to provide occupational medical examination services and related social benefits to surviving migrant ex‑gold miners in Southern Africa, the evidence is that they continue to have difficulty accessing these [18–20]. While extensive research has addressed the risks of silicosis and TB related experiences among migrant miners across Southern Africa [5–7, 9–11, 21], to our knowledge there is only one formal report that has investigated the situation of Malawian ex‑migrant gold miners in accessing unpaid or unclaimed benefits, mainly pension and provident fund related [14]. Our study aimed to fill a gap by drawing on published and unpublished material and interviews with ex‑miners as well as key informants to explore their experiences with regard to health services and benefits for occupational lung disease.

### Key concepts defined

In this report, ‘ex‑migrant miners’ refer to those who had travelled to South African gold mines for work. Occupational health services (OHS) refer to those services to which Malawian citizens are entitled under Malawi’s Occupational Safety, Health and Welfare Act or the Malawian Workers’ Compensation Act [22, 23]. This does not include services specifically for migrant ex‑gold miners such as the provision of benefit medical examinations (BMEs) under South African compensation legislation. The latter is the Occupational Diseases in Mines and Works Act (ODMWA), which provides for BMEs [7, 24, 25]—diagnostic evaluations including physician consultation, chest x‑ray and spirometry for occupational diseases listed in the Act. In this context, these are silicosis, silicotuberculosis and post‑TB impairment, plus some other listed diseases. Claims are adjudicated at the Medical Bureau for Occupational Diseases in Johannesburg. ‘Social benefits’ broadly include specialized health services and compensation for occupational lung disease, as well as unclaimed or unpaid funds and gratuities to which ex‑migrant gold miners may be entitled under past provident or pension funds or other arrangements linked to their mining employment in South Africa [14, 26].

While the focus of this article is on silicosis and occupational TB, chronic employment‑related ill‑health among ex‑gold miners covers a wider spectrum. This includes chronic obstructive pulmonary disease (COPD) attributable to silicosis, TB and general mine dust [27], noise‑induced hearing loss, psychosocial distress [28], permanent disability due to work injuries [29] and elevated HIV risk associated with migrancy [5].

## Objectives

Our primary objective was to describe the barriers facing Malawian ex‑gold miners who worked in South Africa’s gold mines to accessing social benefits related to occupational lung disease, including occupational health services and BMEs. Our secondary objective was to make actionable recommendations to improve access to these services and related benefits.

## Methods

### Data collection

This qualitative study synthesizes available published data and background context, with new community observations and semi‑structured interviews. We triangulated the data we obtained from articles, reports and government documents describing the social benefits systems and relevant barriers with our analysis of the information from interviews and observations. We utilized the 32‑item checklist, consolidated criteria for reporting qualitative research (COREQ), to guide our methods of reporting [30].

A semi‑structured interview guide was used to elicit ex‑gold miner perceptions, as well as key informant perspectives. Interviews with informants were held (in English) in person at their workplaces or virtually online. Ex‑miner interviews were in person at the Ex‑Miner Association of Malawi (EMAM) facility and conducted in the participants’ language of choice (English or Chichewa). Interview questions and subject guides were used to capture data on ex‑miner and informant experiences with the benefits process, ex‑miner quality of life and perspectives on social benefits in Malawi. The same research assistant (RH) conducted all interviews with a translator. Interviews were audio recorded and transcribed verbatim using the Otter.ai software. The interviews each took approximately 15–45 minutes. Miner quotes under Findings are presented in the third person. No repeat interviews were carried out, and only team researchers had access to all data.

### Participants and recruitment

The interviews took place in Blantyre, Malawi, in June 2024. Participants were ex‑migrant miners living in Blantyre and key informants with responsibilities or interests related to the research questions, i.e. one healthcare provider, two government officials and two NGO personnel. Participants were identified using purposive sampling through local networks (of authors YM and KK). Following the initial sampling, snowball sampling was applied for additional participants. Participants were approached by collaborating local partners, such as EMAM, by phone to organize the interviews and transport. No participants declined after initial recruitment, and no participants were excluded. Stakeholders were approached by local networks (YM and KK) and recruited via email and phone. Key informants were selected from relevant sectors: healthcare, government and NGOs. Our authorship team also included two Malawian healthcare providers, both familiar with the state sector and a South African practitioner with considerable experience related to migrant miners’ access to social and health services. This allowed us to reach saturation in our understanding of the situation with a small number of additional key informants.

Interviewees who were ex‑gold miners were in various states of health, with most participants having ongoing health issues. Many ex‑miners are ill, elderly, deceased or unable to be contacted due to a lack of communication channels. We aimed to recruit as many participants as possible and who were feasibly able to be mobilized. Ex‑miners who were interested and able to participate were recruited by EMAM, with no further study withdrawals after being recruited. Participants were approached after being identified as ex‑migrant miners from South Africa, using existing records of EMAM. Participants were not pre‑selected based on having sought help or compensation or being unwell, although, as consistent with purposive sampling, an effort was made to ensure that we had a range of ex‑gold miners, including some who were thought to have been having health concerns, to ensure that their experience and perspective would be included. Each ex‑miner was compensated $25.00 USD as re‑reimbursement for travel expenses, airtime and opportunity costs. Interviews continued until saturation was reached for this location and population.

### Data analysis and management

A phenomenological approach was taken to frame findings and identify patterns/themes within data, using the NVivo 12 software (Lumivero, Denver, USA, 2018). All data have been managed and stored on secure servers and will be retained for at least five years, then securely destroyed. Direct access to data was available to all authors, with all files being password‑protected.

### Ethics approval

Study approval was obtained from the University of British Columbia (UBC) Behavioural Ethics Board (H24‑01335) and the Malawi National Health Sciences Research Committee (24/08/4473). Written consent was obtained at the interview in Malawi. Participants were informed about the study’s purpose, the nature of their involvement and their rights. All data are confidential, have been de‑identified with a unique study ID number and are reported in anonymized form. Results from this work were made available to participants and partners who requested updates on progress.

## Findings

We interviewed 14 ex‑gold miners and five key informant stakeholders (two government officials, two NGO representatives, one healthcare provider). Ex‑miners had a mean age of 67.4 years (range: 62–75) and a mean past mining service of 10.53 years (range: 2–22). Four major themes were produced from the data regarding the situation of migrant ex‑miners in Malawi: (i) inaccessibility of occupational medical examination services in Malawi, (ii) barriers to South Africa administered BME and social benefits processes, (iii) lack of awareness and communication and (iv) poor socioeconomic circumstances of migrant ex‑miners and their families. Each is discussed below.

### Inaccessibility of occupational medical examination services for ex‑gold miners in malawi

Healthcare in Malawi is provided by public, private and non‑profit private sectors with the public health sector being the largest provider, offering free services through the Essential Health Package (EHP) [31]. As mentioned earlier, OHS legislation in Malawi does not cover migrant ex‑miners. Such individuals diagnosed with silicosis or TB have generally been referred to the public healthcare system for investigation and management as appropriate.

Ex‑migrant miners can also access primary‑level mobile diagnostic units that are taken into communities to screen ex‑gold miners for occupational diseases such as TB, as well as tertiary OHS departments set up in the four Malawian central hospitals which receive patients referred from the primary level or direct paying services [31]. The establishment of OHS centres started in 2022, followed by the creation of the Directorate of Occupational Health Services within the Ministry of Health (MOH) in 2024 [32, 33].

However, there remains a lack of access to these services by ex‑miners. Malawi’s public health system is restricted by inadequate resources and funding. There is also a limited focus on ex‑migrant miners’ needs and a lack of awareness among both ex‑miners and stakeholders of available services.

*Our healthcare system is already underfunded. So, if you go to each and every programme within the system you will find that it’s underfunded. (P04, Stakeholder).*

*For ministries and government entities to implement their policies, they need to be funded to do so. (P19, Stakeholder).*

*Just the sheer number of places that can actually do this, beyond the human resources, the places where you can actually have the equipment, the spirometers, the x‑rays, the audio booths, whatever it is, is also limited. (P18, Stakeholder).*

Overall, despite the Malawian government’s positive intentions and current policies, health services suffer from a lack of systemic state funding, supplies and equipment, aggravated in this context by the remoteness and scattered locations of ex‑miners. Stakeholders expressed frustration at the limited channels to provide care for ex‑miners, whether local or ex‑migrant, owing to these resource constraints. This frustration extended to perceptions of a lack of medical expertise, training and capacity in occupational health, in public and private facilities, all of which limited the necessary expansion of an OHS workforce:

*Having a large enough pool of people who pass that training and are confident and have practice is lacking. (P18, Stakeholder)*.

*With our public health care centers, we don’t have specialists dealing with [these] kinds of [occupational lung] diseases (P04, Stakeholder).*

### Barriers to BMEs and social benefits processes in south africa

While Malawian legislation encompasses rights for migrant ex‑gold miners as *citizens* of Malawi, South African legislation covers responsibility regarding occupational lung disease for this population as *ex‑employees*. This separation causes confusion for both ex‑gold miners and stakeholders. Further, while South African gold mining companies historically relied on cross‑border migration to provide cheap labour and continue to pay levies into the ODMWA established compensation fund, the responsibility for the *administration* of examinations and occupational lung disease benefits lies with the South African government [24, 25]. Further, while the Act provides for reporting of ‘suspected’ occupational lung disease by South African medical practitioners and provides for BMEs by such practitioners to be accredited under ODMWA, there is no matching cross‑border arrangement applicable to migrant ex‑miners.

Another hurdle for migrant ex‑gold miners is the narrow eligibility criteria for occupational lung disease compensation. These are based on certified employment in ‘risk work’ (dusty jobs) in a South African mine covered by ODMWA. In the case of compensation for post‑TB lung impairment, proof is required that the TB was diagnosed while working in the industry or within a year of exiting the mines. Despite the burden of sub‑radiological silicosis and silica dust load retained in the lung, TB occurring after this period [7, 12], whether first episode or recurrent, is not compensable.

In addition to medical eligibility, the administrative process is complex and potentially costly—including the acquisition of certified fingerprints, a certified occupational history, previous TB test results—and for dependents, a marriage certificate (uncommon in rural communities) [7, 21, 24, 25]. In the case of deceased claimants without an autopsy, for the beneficiaries to be eligible for compensation, the death certificate has to list the occupational lung disease as the primary cause of death. This is seldom done. ODMWA uniquely provides for the heart and lungs of deceased miners to be examined for purposes of post‑mortem compensation. This requires prosection and transport of the heart and lungs to a pathology laboratory in Johannesburg [21]. However, there is limited uptake from black ex‑miners generally, owing to the difficulties of finding qualified local prosectors and of tissue transport across borders, as well as a lack of miner knowledge of this system while alive and of family consent after death [21, 24, 25].

In living miners, an accredited physician must be found or contracted to conduct a BME and submit the application with medical documents to the Medical Bureau for Occupational Diseases in South Africa. If the applicant is deemed eligible, the Bureau refers the claim to the office of the Compensation Commissioner for Occupational Disease, who, after the receipt of the remaining documents, makes payment. This two‑stage separation of responsibilities presents further administrative barriers [7, 20, 21]. The benefits process was described as arduous and inefficient, involving repetitive cycles without clarity or results, and thus creating mistrust:

*They have tried, but they’re still just being considered cheated [by the Malawian government]. Because they will be told that maybe next week you will be getting your funds, but then they’ll see that nothing is happening. (P09, Ex‑Miner).*

A stakeholder described the negative view of current benefits processes among ex‑miners:

*I feel like the requirements for ex‑miners to access these benefits, sometimes they feel like they’re just there to make sure that the ex‑miner cannot access the benefits(P04, Stakeholder)*.

At an inter‑country level, attempts to mitigate the impact of mining in propagating TB in Southern Africa have resulted in a number of regional coordination and internationally funded programmes in the Southern African Development Community [7, 34, 35]. One such initiative is ‘TB in the Mining Sector in Southern Africa’ (TIMS), funded by the Global Fund to Fight AIDS, Tuberculosis and Malaria and covering ten countries [7, 36]. Phase 1 and 2 of the project included the TB screening of approximately 15,300 individuals in Malawi—miners, ex‑miners and family and community members [36]. Since to our knowledge, no report of the TIMS examination findings has been publicly released, it is not possible to determine whether migrant ex‑miners were included in the programme, and whether silicosis was screened for (as was the case in other countries) [11].

A parallel development affecting miner benefits was the establishment of the Tshiamiso Trust following the settlement in 2018 of a class action lawsuit for silicosis and TB [37, 38]. This followed a South African constitutional court judgement which overturned the legal status quo which had prevented mine employees from suing their employers for occupational lung disease. The court’s finding means that South African mining companies can be held legally accountable for negligence in failing to prevent hazardous working conditions. The Trust is in the process of tracing ex‑gold miners and conducting BMEs. The latter are privately organized and funded by the Trust. However, the settlement leaves intact the current statutory system and will expire in 2030. How many Malawian ex‑gold miners will benefit from the Trust remains to be seen.

A major concern for ex‑migrant miners is the recovery of unpaid social benefits [14, 17, 26]. Compensation for occupational lung disease forms a small part of this, with a 2018 source indicating 402 certified but unpaid claims to Malawians dating from 1973 [39]. The bulk of unpaid benefits, in many cases with untraceable beneficiaries, are pension and provident fund payouts, which are managed by private entities in South Africa. Ex‑gold miners interviewed also mentioned gratuities and long‑service awards from their employment in the South African gold mines. They blame both the Malawian and South African governments for their failure to receive what they believe is owed to them under these benefit systems:

*The government was responsible for them when they were going to the mines…it was ordered from [the Malawian] government and the South African government (P02, Ex‑Miner)*.

Currently, ex‑migrant gold miners in Malawi, through their association EMAM, are pursuing legal avenues in negotiation with the Malawian Government. These concern the long‑standing conflictual issues between the government and ex‑miners regarding their original recruitment contracts and employment‑related gratuities associated with those contracts [14]. The ex‑migrant miner’s mistrust towards the Malawian government is evident:

*It’s in the hands of the lawyer who is going to be presenting [the case], as the government has failed them…they have failed to speed up the process for them to get their compensation (P08, Ex‑Miner).*

### Lack of awareness and communication

The division of responsibility between the governments of South Africa and Malawi has resulted in confusion and misinformation among ex‑gold miners concerning their rights and entitlements and the jurisdictions of responsibility for the benefits process and services:

*He was never aware of the process that one has to undergo for him to get compensated. (P08, Ex‑Miner).*

Stakeholders have found that the misinformation within ex‑gold miner communities is the result of limited available public information, remoteness of communities, and a lack of communication channels, with community leaders as the sole messengers for their communities. Efforts to disseminate information were seen as inadequate, leaving miners feeling excluded and disempowered. However, even after miners understand the complicated process, they must still make a choice as to whether a claim is worth making depending on their chances of getting compensated; even after participating in the lengthy benefits process, they are not guaranteed compensation.

### Poor socioeconomic circumstances of ex‑migrant miners and their families

Ex‑gold miners reported dangerous conditions within the mines that led to many physical injuries and psychological trauma:

*Some people, they were crushed with stones. Every time he was going to the mines, he just had to pray, ‘I should come back alive’ (P14, Ex‑Miner).*

Ex‑gold miners attributed their poor physical conditions to their years of mining, including chronic cough, chest pains, asthma, TB, strokes, liver disease and benign growths. Ex‑gold miners have found themselves with significant financial hardships due to their health problems, including injury‑related disabilities and chronic ill‑health. This burden has prevented their participation in the formal economy, forcing reliance on other sources of income such as selling coal, plastic bags or repairing electronics.

Male ex‑miners are traditionally the breadwinners for their families and active members of their communities. Families were deeply affected by their loss of employment and failure to secure any financial redress. Ex‑miners were now seen as unable to provide food and education for their children and overall financial support. This has led to the financial and caregiving burden falling onto the families:

*Since the man is failing to provide due to a lack of financial resources, the woman is trying to get into some other sexual activities so she can provide money (P10, Ex‑Miner).*

Ex‑gold miners reported poor living conditions such as lack of clean water, sanitation and basic necessities such as soap and food. For instance, one ex‑miner described washing their clothes only twice a year:

*Family members are very worried. There was a situation that right now, he is even failing to bathe his son (P09, Ex‑Miner).*

One ex‑miner described their struggle:

*It is very difficult. My survival is very difficult. Sometimes I have some relatives, they give me food (P02, Ex‑Miner).*

These hardships have contributed to worsening health, psychological distress and social and community alienation:

*Lately, they have been facing a lot of challenges that have unfortunately led to some people committing suicide (P09, Ex‑Miner).*

Given Malawi’s resource constraints, stakeholders and ex‑miners highlighted the need for local support by NGOs or external support from international partners. Stakeholders emphasized that multi‑sectoral support was needed to expedite the benefits process and provide service access for migrant ex‑miners. Ex‑miners expressed the view that support should focus primarily on providing basic necessities for everyday survival (e.g. food, hygiene, job creation). One stakeholder noted that while policy change was necessary, the priority was assisting ex‑miners:

*The great majority are going to end up in with these catastrophic constant issues about poverty and exacerbating poverty. I think that that’s going to be the main issue (Stakeholder, P18).*

## Discussion

Our study is the first to explore the plight of Malawian ex‑migrant miners from the South African gold mines with regard to occupational lung disease related social benefits. Our findings have applicability to other countries from which migrant gold miners were drawn to work in these mines, allowing for some local specificities. Individual‑level barriers include the passage of time since last employment and lack of knowledge or misinformation about the process. System‑level barriers include lack of the necessary cross‑border arrangements, underfunding and functional inefficiencies leading to communication and payment delays. As a result, regardless of country of origin, many ex‑gold miners continue to suffer poor health, financial stress and inaccessibility of appropriate health services or statutory examinations for their occupational lung disease [5, 7, 10, 18–21, 24, 25, 39]. What is unique about Malawi is the passage of over 30 years since the last central recruitment and the fact that a Malawian government agency was directly involved in recruitment [13, 14]. Consequently, an argument can be made for additional accountability on the part of the Malawian government.

While occupational health services, policies and government intent are present in Malawi, they are concentrated on domestic miners, but with regulation and enforcement often deferred, creating a gap between policy and implementation. Regarding occupational lung disease compensation and services for migrant ex‑miners, there remains fragmentation—through inefficient collaboration between the multiple processes and actors, e.g. the Malawian Ministry of Health, the Directorate, the Ministry of Labour, and as described previously, South African entities. The new Directorate in Malawi [33] has an opportunity to mitigate this problem by taking up some of the responsibility for ex‑migrant miners. By building on existing programmes/services, the Directorate could act as a central hub to increase collaboration between actors, promoting access to BMEs for ex‑migrant miners and awareness of OHS and benefits.

Despite the existence of several instruments for regional cooperation, the lack of specific cross‑country agreement is a crucial barrier to accessing social security by ex‑miners and efforts to monitor these different processes [26]. Under ODMWA, ex‑miners if not already maximally compensated, are entitled to a BME every two years. While BME capacity exists in Malawi, there is no working mechanism currently for their accreditation and reimbursement by ODMWA agencies. The mechanism of direct cross‑country payment for these examinations needs to be resolved with the compensation agency in South Africa to ensure BME access and processing. There is also a need to investigate the status of the unpaid ODMWA claims from Malawian ex‑miners referred to earlier [39].

Within Malawi, professional occupational health training needs to include standardized training for BME examination that meets South African standards and that is recognized in both countries. In this context, emerging technologies could serve as adjunctive tools to serve ex‑gold miner occupational health and health equity. Specifically, artificial intelligence and computer‑assisted CXR detection to support efficient compensation claim processes for ex‑miners in Southern Africa would contribute to healthcare delivery in resource‑constrained settings [40, 41]. This could be done through extending existing activities using mobile CXR units and ultraportable x‑ray systems to reach remote ex‑migrant miner communities [42].

While information about TB has been disseminated to some ex‑miner communities in the past, there is a need to step up efforts to inform ex‑migrant gold miners about specific benefits and OHS to which they may be entitled, including which institutions are responsible and how to access these services. This information needs to be appropriate to community literacy, education and language levels, through radio advertisement, door‑to‑door contacts, infographics and local language posters.

Finally, as noted by other authors, arrangements between labour‑sending governments and mines subordinated workers’ rights to economic benefits derived from cheap labour [43]. One outcome was the transfer of the ill‑health burden to the home country’s health system after the miners returned home. In this respect, miners’ occupationally related morbidity and mortality have been under‑recorded, leading to difficulties in projecting liability and individual claiming of social benefits today [7, 43]. A historical anomaly is that while statutory compensation payments are made from levies on the mining companies, the costs of administering the statutory compensation system are borne by the South African government [25]. The annual administrative budget for this purpose was approximately R64 million in 2024 (US$3.3 million, 8 March 2025) [44]. This is comparable to the annualized administrative budget of the Tshiamiso Trust of R70.4 million p.a. ($US 3.6 million) (total cap R845 million spendable over 12 years) and paid for entirely by the industry’s settlement with mine workers [45].

There is therefore a case for the South African gold mining industry to contribute financially to the further development and sustainability of the service platform for foreign ex‑miners, providing the capacity to enter, track and verify claims, examination results and treatment history, and to avoid the transfer of the financial burden of administration to home governments. There is a precedent in the mining industry’s recent financial and operational support for the Compensation Commissioner for Occupational Disease and the Compensation Claims Management System, a digital platform used by the Commissioner and by the Tshiamiso Trust [39, 46].

As of 2020, there was no reliable official database of migrant ex‑miners in Malawi [14]. Reliance purely on TEBA records is likely to be insufficient [7]. An important step would therefore be the linkage of this group into the digital platform. This would include finding ways to assist those who may no longer have official documentation to acquire such [14].

Ex‑miners are uncontestably owed eligibility examinations for monetary compensation through existing systems such as ODMWA and the Tshiamiso Trust. However, these existing systems, and particularly the question of whether current compensation is enough to address structural injustice, need to be critically examined [28, 47]. For example, only 27% of compensation claims submitted to the Medical Bureau for Occupational Diseases in recent years have been found to be compensable [39]. With regard to those who were compensable, the inflation‑adjusted value of lung disease compensation under ODMWA declined sharply from 1973 to 1990, recovering only partly for black miners with the removal of racial discrimination after 1993 [24].

Evidence regarding the socioeconomic burden carried by migrant ex‑miners has generally been lacking—a gap in itself. All ex‑gold miners interviewed in this study discussed their need for support for the basic necessities required for everyday survival, with stakeholders corroborating this opinion. Given that there is no social assistance specifically for this purpose, ex‑goldminers blame the Malawian government, especially since no aid is coming from South African institutions. Since compensation for occupational disease is bound by strict eligibility requirements and largely inadequate for long‑term financial support [47], the argument can be made that ex‑miners are owed relief aid in the form of support programmes covering food and the amenities of daily life as a matter of social justice. A list of potential programmes has previously been advertised with regard to Lesotho by TEBA Ltd., now a private company contracting to the mining industry [48]. These included programmes and projects covering home‑based care, TB management, spinal cord injured mine worker care, silicosis tracing, portable skills training, food security and infrastructure development.

This study was limited by a relatively small number of interviews (*n* = 14) with ex‑miners, all of whom were from the Blantyre region. Given that many ex‑miners in this region are old and/or ill and live in remote locations this was the number that was feasible to mobilize. However, we believe that the interviews with ex‑miners, combined with the key informant interviews, were able to reach saturation with respect to understanding the challenges and illuminating the need to examine the role played by the mining sector when considering accountability.

Coordinated efforts between the Malawian and South African governments, local NGOs and international organizations are needed to find means to provide relief for ex‑miners. However, there is also a need to seek ways to hold employer mining companies accountable for the ill‑health and related socioeconomic burden affecting this and other ex‑mining populations in Southern Africa.

## Figures and Tables

**Table:** 

TB	Tuberculosis
EMAM	Ex‑Miners Association of Malawi
NGO	Non‑Governmental Organization
OHS	Occupational Health Services
ODMWA	Occupational Diseases in Mines and Works Act (South Africa)
BME	Benefit Medical Examination
TIMS	TB in the Mining Sector in Southern Africa

## Additional Files

The additional files for this article can be found as follows:

Supplementary Table 1. The 32‑item checklist: consolidated criteria for reporting qualitative research (COREQ) to guide our methods reporting as applied within our study.

Supplementary Table 2. Codes and themes.
