# Supplementary material for: Unmet Occupational Health Needs of Malawian Ex-Miners from the South African Gold Mines
Source: Ann Glob Health. 2025 Jun 5;91(1):26. doi: 10.5334/aogh.4680 (PMC12143258; doi:10.5334/aogh.4680)
Supplement: Supplementary Table 1. — The 32‑item checklist: consolidated criteria for reporting qualitative research (COREQ) to guide our methods reporting as applied within our study. [file agh-91-1-4680-s1.pdf]

Supplementary Material

Table 1. The 32-item checklist: consolidated criteria for reporting qualitative research (COREQ) to guide our methods reporting as applied within our study.

Checklist item	Application within our study
Which author/s conducted the interview or focus group?	RH
What were the researchers' credentials?	MPH (RH); MD or MBBS (AY, RE, YM, KK), PhD (RE, JS, KK)
What was their occupation at the time of the study?	Master's student (RH); Professors (AY, JS, KK), Emeritus Professor (RE), occupational health practitioner (AY, RE, YM)
Were the researchers male or female?	Female (RH, AY), Male (RE, JS, YM, KK)
What experience or training did the researchers have?	Prior qualitative data collection and clinical health research experience, decades of mixed methods research experience.
Was a relationship established prior to study commencement?	Two of the authors are Malawian with strong ties; a relationship was established for the other team members prior to study commencement through Malawian team members communicating with local organizations in Blantyre.
What did the participants know about the researcher? e.g. personal goals, reasons for doing the research	Participants were aware of the researcher's role, purpose of the study and their participation/rights.
What characteristics were reported about the interviewer/facilitator? e.g. Bias, assumptions, reasons and interests in the research topic	The original submission of the article noted the expertise, experience and positionality of team members, but a reviewer felt that this did not belong in the article, so only roles in recruitment are now noted.
What methodological orientation was stated to underpin the study?	Phenomenological approach
How were participants selected? e.g. purposive, convenience, consecutive, snowball	Purposive and snowball sampling. The process is described in depth.
How were participants approached? e.g. face-to-face, telephone, mail, email	By phone from EMAM and local team members
How many participants were in the study?	14 ex-miners and 5 key informants
How many people refused to participate or dropped out? Reasons?	None
Where was the data collected? e.g. home, clinic, workplace	Ex-miners were interviewed at the EMAM office facility and informants were interviewed at their workplaces (e.g. hospital) or online over Zoom.
Was anyone else present besides the participants and researchers?	Translator
What are the important characteristics of the sample? e.g. demographic data, date	The cohort is described in the text; interviewees were all male ex-miners of advanced age. The demographics of key informants are not described to preserve confidentiality given the limited number of personnel in each category.

Were questions, prompts, guides provided by the authors? Was it pilot tested?	Interview guides were reviewed and approved by EMAM (the NGO representing the ex-miners) and team members.
Were repeat interviews carried out? If yes, how many?	N/A
Did the research use audio or visual recording to collect the data?	Audio recording was used
Were field notes made during and/or after the interview or focus group?	Field notes were made during and after interviews and observations.
What was the duration of the interviews or focus group?	15-45 minutes
Was data saturation discussed?	Yes.
Were transcripts returned to participants for comment and/or correction?	Given the logistical difficulties in transportation of elderly and ill interviewees in this rural low-income region, this was not done. However, we felt that the positionality and triangulation process used assured considerable accuracy.
How many data coders coded the data?	One (RH), in discussion with team members.
Did authors provide a description of the coding tree?	Yes (Supplementary Material, Table 2)
Were themes identified in advance or derived from the data?	Derived from the data, and informed by existing scholarship
What software, if applicable, was used to manage the data?	Nvivo 12
Did participants provide feedback on the findings?	Not directly. See note above regarding transcripts.
Were participant quotations presented to illustrate the themes / findings? Was each quotation identified? e.g. participant number	Yes
Was there consistency between the data presented and the findings?	Yes
Were major themes clearly presented in the findings?	Yes
Is there a description of diverse cases or discussion of minor themes?	Yes
