# Supplementary material for: Unmet Occupational Health Needs of Malawian Ex-Miners from the South African Gold Mines
Source: Ann Glob Health. 2025 Jun 5;91(1):26. doi: 10.5334/aogh.4680 (PMC12143258; doi:10.5334/aogh.4680)
Supplement: Supplementary Table 2. — Codes and themes. [file agh-91-1-4680-s2.pdf]

Table 2. Codes and themes

Major theme	Codes	Subcodes	Participant
Inaccessibility of occupational medical examination services in Malawi	Support required	Access, benefits, required programs and policies	Ex-miner
	Lack of health system resources and funds		Key Informant
	Lack of access	Remoteness/transportation, lack of healthcare services, medical affordability/shortage	Ex-miner
	Health practitioner capacity and training		Key Informant
Barriers to South Africa administered BME and social benefits processes	Industry systems and organizations	Accountability, lack of government intervention, systemic reform, policy versus implementation	Ex-miner, Key Informant
	Health system barriers		Key Informant
	Compensation process	Benefits criteria, beneficiaries, document apprehension	Ex-miner, Key Informant
Lack of awareness and communication	Lack of awareness	Compensation process, health risks of mining	Ex-miner, Key Informant
Poor socioeconomic circumstances of migrant ex-miners and their families.	Support required	Basic necessities, finances	Ex-miner
	Mining conditions	Death, mining accidents, PPE, safety	Ex-miner
	Emotional impact		Ex-miner, Key Informant
	Community impact		Ex-miner, Key Informant
	Family impact		Ex-miner
	Health conditions		Ex-miner, Key Informant
	Poor quality of life	Daily survival, lack of food	Ex-miner, Key Informant
	Protective factors	Current policies, positive government aid, government involvement	Ex-miner, Key Informant
